# Iron Oxide and Silicon Nanoparticles Modulate Mineral Nutrient Homeostasis and Metabolism in Cadmium-Stressed *Phaseolus vulgaris*

**DOI:** 10.3389/fpls.2022.806781

**Published:** 2022-03-21

**Authors:** Lyubka Koleva, Aisha Umar, Nasim Ahmad Yasin, Anis Ali Shah, Manzer H. Siddiqui, Saud Alamri, Luqman Riaz, Ali Raza, Talha Javed, Zunera Shabbir

**Affiliations:** ^1^Department of Plant Physiology and Biochemistry, Agricultural University, Plovdiv, Bulgaria; ^2^Institute of Botany, University of Punjab, Lahore, Pakistan; ^3^Senior Superintendent Garden, RO-II Office, University of Punjab, Lahore, Pakistan; ^4^Department of Botany, Division of Science and Technology, University of Education, Lahore, Pakistan; ^5^Department of Botany and Microbiology, King Saud University, Riyadh, Saudi Arabia; ^6^Department of Environmental Sciences, University of Narowal, Punjab, Pakistan; ^7^Key Laboratory of Ministry of Education for Genetics, Breeding and Multiple Utilization of Crops, Centre of Legume Crop Genetics and Systems Biology/College of Agriculture, Oil Crop Research Institute, Fujian Agriculture and Forestry University (FAFU), Fuzhou, China; ^8^College of Agriculture, Fujian Agriculture and Forestry University, Fuzhou, China; ^9^Agronomy, Horticulture, and Plant Science Department, South Dakota State University, Brookings, SD, United States

**Keywords:** growth, potassium, polyamines, antioxidant, stress, cadmium

## Abstract

The application of nanoparticles (NPs) has been proved as an efficient and promising technique for mitigating a wide range of stressors in plants. The present study elucidates the synergistic effect of iron oxide nanoparticles (IONPs) and silicon nanoparticles (SiNPs) in the attenuation of Cd toxicity in *Phaseolus vulgaris*. Seeds of *P. vulgaris* were treated with IONPs (10 mg/L) and SiNPs (20 mg/L). Seedlings of uniform size were transplanted to pots for 40 days. The results demonstrated that nanoparticles (NPs) enhanced growth, net photosynthetic rate, and gas exchange attributes in *P. vulgaris* plants grown in Cd-contaminated soil. Synergistic application of IONPs and SiNPs raised not only K^+^ content, but also biosynthesis of polyamines (PAs), which alleviated Cd stress in *P. vulgaris* seedlings. Additionally, NPs decreased malondialdehyde (MDA) content and electrolyte leakage (EL) in *P. vulgaris* plants exposed to Cd stress. These findings suggest that stress alleviation was mainly attributed to the enhanced accumulation of K^+^ content, improved antioxidant defense system, and higher spermidine (Spd) and putrescine (Put) levels. It is suggested that various forms of NPs can be applied synergistically to minimize heavy metal stress, thus increasing crop production under stressed conditions.

## Introduction

Mining operation, industrial waste, and pollutants emitted during agricultural and industrial operations (Palansooriya et al., 2020; Raghib et al., 2020) release substantial quantities of toxic heavy metals (As, Cd, Hg, Pb) into the environment. The quantity of harmful heavy metals in the soil environment has steadily increased over time, which produces negative effects on plant growth and their developmental process, which is a great challenge for the sustainability of agriculture (Afzal et al., 2019). Among the numerous heavy metals, Cd content in the environment has risen owing to excessive use of chemical fertilizers and pesticides besides mining sources (Seshadri et al., 2016; Manzoor et al., 2019). Although the concentration of Cd reported in the environment is low, it is considered extremely toxic due to its higher mobility in media and living cells (Rahman and Singh, 2019; Kaya et al., 2020a), which is raising concerns for agriculture. It was reported that Cd exposure reduces seed sprouting, root elongation, shoot development, and the number of leaves per plant (Kaya et al., 2020b; Seifikalhor et al., 2020). In addition, it also results in the overproduction of reactive oxygen species (ROS) (Moradi-Marjaneh et al., 2019), which influence plant physiological, biochemical, and molecular characteristics (Raza et al., 2020). Plants can avoid, tolerate, and immobilize heavy metals in soil (Kumar and Prasad, 2018; Mei et al., 2020) by activating signaling molecules that regulate ROS production, phytohormone synthesis, and calcium calmodulin pathways (Bali et al., 2019). The activated pathways include the production of nitric oxide (NO), glutathione, phytohormones, and antioxidant enzymes (Dias et al., 2019; Rady et al., 2019), which lead to Cd chelation and reduced oxidative damage.

Nanoparticles (NPs) with a size of 100 nm exhibit unique properties such as increased reaction site, high surface activity, better catalytic efficiency, and unique magnetic characteristics (Yang et al., 2017, 2018; Wang et al., 2019), which have assisted in agriculture promotion by alleviating abiotic stress in plants (Adeel et al., 2019). Many researchers have reported that NPs improve seed germination, rhizome development, and quality of crops cultivated in stressed conditions (Ghafariyan et al., 2013; Palchoudhury et al., 2018; Kah et al., 2019). Nanotechnology research offers a new pathway for soil pollution remediation (Liu et al., 2021), since it has the potential to enhance plants’ antioxidative defense systems, thus reducing the bioaccumulation of ROS in plants (Usman et al., 2020; Wang et al., 2020). As documented, NPs when applied in soil (Li et al., 2021) or sprayed *via* foliar (Toumey, 2020), results in a reduction of Cd and Pd toxicity in rice seedlings (Hussain et al., 2020). It was observed that the Si improved hydraulic conductivity in applied plants through enhancing K^+^ concentration in xylem sap resulting in improved xylem hydraulic conductivity and osmotic gradient (Chen et al., 2016). Similarly, exogenously applied Fe modulates K^+^ uptake and translocation in crop plants (Okturen Asri and Sonmez, 2014).

Potassium is a crucial phytonutrient required by plants for the maintenance of growth (Jaiswal et al., 2016), stomatal conductance, gaseous exchange (Hasanuzzaman et al., 2018), photosynthate production, and antioxidative defense system (Xu et al., 2020). Approximately 10% of plant biomass is composed of K, which not only acts as a co-factor for various enzymes involved in photosynthesis and protein stabilization, but it also hinders Cd-translocation and escalates the production of crucial amino acids, carbohydrates, and nitrogenous compounds. Potassium improves plant stress tolerance by regulating the biosynthesis of polyamines (Karimi, 2017). Polyamines regulate physiochemical procedures of plants under normal conditions and also induce modification in the expression level of stress-responsive genes besides detoxifying metals by vacuolar compartmentalization (Spormann et al., 2021).

Considering the background information, we hypothesized that the synergistic effect of NPs can mitigate Cd stress; and regulate polyamine synthesis, K^+^ metabolism, and antioxidant enzymes. Therefore, the objective of the present study was to explore the individual and combined role of IONPs and SiNPs application in mitigation of Cd toxicity through regulation of K^+^ metabolism and antioxidant enzymes in *P. vulgaris* exposed to Cd-contaminated conditions.

## Materials and Methods

A total of 85 soil samples were collected (0–30 cm) from an agricultural field (2-ha area) in the vicinity of the campus site. The soil was sieved through a 4 mm mesh to remove plant parts, debris, then thoroughly mixed and conditioned for 1 week at 35% of water holding capacity (WHC) before the experiment. The physicochemical properties of the soil such as pH, electrical conductivity (EC), organic matter, and metal content (Cd, Zn, Fe, Ni, Pb) were measured using standard protocols (Bouyoucos, 1962; Page et al., 1982). The physicochemical properties of the soil were as follows; pH (7.13), EC (1.87), and organic content (0.45%). The total concentration of Cd, Zn, Fe, Ni, and Pb in soil was quantified as 5.87, 34.98, 37.91, 13.28, and 35.18 mg/kg, respectively. The soil was contaminated by mixing CdCl_2_. Cadmium concentrations in the soil were kept as 0 mM CdCl_2_, 1 mM CdCl_2_, 1.5 mM CdCl_2_, and 2 mM CdCl_2._ Both IONPs and SiNPs were purchased from Alfa Aesar. A pilot project was carried out to find out the toxic concentration of Cd that affects the growth of *P. vulgaris*. A 10 mg/L IONPs and 20 mg/L SiNPs were found to be effective against selected Cd toxic levels. Iron oxide nanoparticles (IONPs) obtained from Alfa Aesar were having 99% purity, size 15–25 nm, and 4.67 density. Silicon nanoparticles obtained from Alfa Aesar were having 98% purity, size 40–100 nm, and 4.7 density. The solution used during the experiment was prepared using deionized H_2_O.

### Seed Priming With Iron Oxide Nanoparticles and Silicon Nanoparticles

Seeds of *P. vulgaris* were surface sterilized using 2.5% sodium hypochlorite solution for 2 min and then washed with deionized H_2_O to remove chlorophyll (Chl) contents. IONPs and SiNPs were weighed and added in deionized H_2_O followed by ultra-sonication for half-hour and their desired concentrations (IONPs = 10 mg/L) and (SiNPs = 20 mg/L) were achieved. After that sterilized seeds of *P. vulgaris* were soaked in an NP solution. In the case of the control treatment, seeds were treated with deionized H_2_O. In the next step, soaked seeds were dried and stored at 4°C for further experiments.

### Seed Sowing and Greenhouse Conditions

The current study was executed at the university site (32°′N, 74°′E, 236m altitude). Ten seeds were sown in plastic pots of 40 × 45 cm containing 20kg treated soil. These pots were kept in the greenhouse condition with an average temperature of 20/15°C (day/night), average humidity of 50/70% late afternoon/morning, and a natural photoperiod. After germination, thinning was done and 8 plants were retained per pot. Regular weeding was done and plants were watered daily to uphold 70% of the field maximum moisture capacity throughout the growth period.

### Determination of Growth and Leaf Relative Water Content

Plants were uprooted after 40 days and growth characteristics (root and shoot fresh weight, root and shoot dry weight) were estimated. Leaf relative water content (LRWC) values from *P. vulgaris* leaf samples were estimated using the following equation as given by Smart and Bingham (1974);


LRWC(%)=LFW-LDW/LTW-LDW×100

Where LRWC = Leaf relative water content

LFW = Leaf fresh weight

LDW = Leaf dry weight

LTW = Leaf turgid weight

### Estimation of Photosynthetic Pigments

Arnon method (1949) was used for the estimation of photosynthetic pigments. Approximately 100 mg leaf extract was mixed with 80% acetone and centrifuged for 5 min at 10,000 rpm. The optical density (OD) of filtrates was then measured using a spectrophotometer (Hitachi U-2001, Tokyo, Japan).

### Determination of Malondialdehyde Content and Electrolyte Leakage

Malondialdehyde (MDA) content was determined according to the method of Cavalcanti et al. (2004). Briefly, 0.5 g samples were homogenized using mortar and pestle in 4 mL of trichloroacetic acid (TCA) (1% w/v) at 4°C. The homogenous mixture was then centrifuged for 20 min at 12,000 rpm to collect the supernatant. One milliliter of this supernatant was added to 3 mL of reaction mixture containing TCA (20% w/v) and thiobarbituric acid (0.5% w/v). The reaction mixture was incubated at 95°C for half-hour and the reaction was stopped by placing it in an ice bath. The absorbance value of the fraction was measured at 440, 532, and 600 nm.

The method of Blum and Ebercon (1981) was used for the determination of electrolyte leakage. A dry leaf sample (0.2 g) was floated in deionized H_2_O (50 mL) for 24 h at room temperature with shaking. The electrolyte content in the solution was quantified and recorded as CO. After 20 min of boiling the sample, the electrolyte content in the solution was recorded as C1. Electrolyte leakage was measured in percentage as per the following formula:


Relativeelectrolyteleakage=CO/C1×100

### Determination of Antioxidant Enzymes

About 1 g leaf samples were homogenized in a solution of potassium phosphate buffer solution (100 mM), polyvinyl pyrrolidone (1% w/v), ethylenediamine tetraacetic acid (0.1 mM) and triton X-100 (0.5%). The homogenate was filtered through cheesecloth and centrifuged at 18,000 rpm for 20 min at 4°C. The resultant supernatant was used for the estimation of antioxidative enzymes and was stored at –80°C for analysis. The method of Aebi (1974) was used for the determination of catalase (CAT) activity. Briefly, potassium phosphate buffer (50 mM) and plant extract were used in the reaction (3 mL). To start the reaction, 10 mM H_2_O_2_ was added. 1 unit of CAT is defined as the number of enzymes, which release 1/2 of peroxide oxygen from 10 mM H_2_O_2_ solution in 100 s at 25°C. Superoxide dismutase (SOD) activity was measured according to the method of Beyer and Fridovich (1987). The reaction mixture (30 mL) for the determination of SOD was composed of potassium phosphate buffer (50 mM), methionine (9.9 mM), nitroblue tetrazolium (57 mM), and plant extract. The reaction mixture was started by light illumination. One unit of SOD is defined as the enzyme which causes a 50% decrease of SOD inhabitable reduction.

### Determination of Proline Content

To determine proline content, dry leaf samples (0.5 g) were extracted in sulfosalicylic acid (3%) and then filtered (Bates et al., 1973). Afterward, 2 mL of leaf extract was mixed in ninhydrin solution (2 mL) and glacial acetic acid (2 mL). Plant samples were incubated at 100°C for 1 h and cooled in an ice bath. Four milliliters of toluene was vigorously added to the mixture. The absorbance value was calibrated at 520 nm using a spectrophotometer. Proline content was estimated using a standard curve.

### Determination of Cadmium Content

Following Cd treatment, *P. vulgaris* seedlings were rinsed with deionized H_2_O. Seedlings were dried at 80°C for 24 h. The plant material was digested in HNO_3_:HCLO_4_. About 1 g plant sample was homogenized in 50 mM Tris (hydroxymethyl) aminomethane (Tris–HCl), 250 mM sucrose, and 1 mM DL-dithiothreitol. The homogenous mixture obtained was centrifuged at 3,000 rpm for 5 min for the isolation of the cell wall. The supernatant obtained was centrifuged at 20,000 rpm for 45 min. Cd was determined using ICP (ICP-AES, Thermo Elemental, United States) (Wang et al., 2009).

### Determination of Nutritional Content

A flame photometer (Jenway PFP-7) was used for the determination of nutritional content (Mo^+^, Ca^+^, K^+^, Mn^+^) from the digested plant samples. K^+^ content was quantified with the help of a standard solution.

### Determination of Gaseous Exchange

Assessments of gaseous exchange attributes, net CO_2_ uptake, net photosynthesis (Pn), stomatal conductance (Gs), and transpiration rate (E), were performed at 10:00 a.m. at 27°C during the daytime on completely expanded leaves using a portable gas exchange system (Holá et al., 2010).

### Determination of Polyamine Activity

The concentration of hydrogen peroxide produced during polyamine oxidation assisted in the estimation of polyamine oxidase (PAO) activity. The foliage plant sample was mixed with potassium phosphate buffer (0.1 mM at pH 6.5 in an pre-chilled mortar and pestle. The resultant supernatant (0.5 mL) was obtained by centrifugation of this mixture at 10,000 g at 4°C for 20 min followed by mixing with 4-aminoantipyrine/*N*,*N*-dimethylaniline solutions (0.4 mL), 0.2 mL horseradish POD (250 U/mL), and 5 mL of K_3_PO_4_ buffer (100 mM at pH 6.5). For the determination of polyamine oxidase, putrescine (30 mL of 20 mM) was homogenized in enzyme extract to initiate reaction according to Zhao et al. (2004).

### Determination of Nitric Oxide Content

Nitric oxide activity was quantified using the nitric oxide detection kit (Solarbio Life Science, Beijing, China) following the manufacturer’s instructions.

### Estimation of Soluble Protein Content

Leaf samples (1 g) were extracted using KH_2_PO_4_ (4 mL at pH 6.8) following centrifugation at 3,600 rpm for 30 min. The resultant supernatant was collected and a 20 μL aliquot of the extract was collected and homogenized with the Bradford color reagent (1 mL). The spectrophotometer calibration was carried out at 595 nm as per the procedure described by Bradford (1976).

### Statistical Analysis

Data reported in the experiment was mean of 5 replicates. Statistical package XL-STAT was carried out for analysis of variance (ANOVA). Subsequently, Tukey’s test was conducted to determine the significant differences among the values.

## Results

### Effect of Iron Oxide Nanoparticles and Silicon Nanoparticles on Growth Attributes of *Phaseolus vulgaris*

Table 1 shows the role of IONPs and SiNPs, alone or in combination, on the growth of *P. vulgaris* seedlings grown in normal and Cd-contaminated soil. Synergistic application of NPs improved shoot fresh weight and root fresh weight by 39 and 40%, respectively, as compared to IONPs-only treated *P. vulgaris* seedlings grown in non-contaminated soil. When *P. vulgaris* seedlings were exposed to 2 mM CdCl_2_, combined treatment of IONPs and SiNPs increased shoot fresh weight and root fresh weight by 45 and 19%, respectively, as compared to SiNP-treated *P. vulgaris* seedlings grown in Cd-contaminated media. Likewise, combined and synergistic treatments involving NPs enhanced root dry weight and shoot dry weight in *P. vulgaris* seedlings grown in normal and Cd-contaminated conditions (Table 1).

**TABLE 1:** Effect of IONPs and SiNPs on root fresh weight, shoot fresh weight, root dry weight, shoot dry weight, leaf relative water content, and net photosynthetic rate of *Phaseolus vulgaris* grown in different concentrations of Cd.

Treatments	Root FW (g plant^–1^)	Shoot FW (g plant^–1^)	Root DW (g plant^–1^)	Shoot DW (g plant^–1^)	LRWC (%)	Net Photosynthetic rate (μmol m^–2^ s^–1^)
Cd0 + IONPs	4.21 ± 0.45ab	25.12 ± 1.89b	0.33 ± 0.0021b	2.76 ± 0.54bc	82 ± 6.76	1.98 ± 0.35cd
Cd0 + SiNPs	3.76 ± 0.37cd	23.18 ± 1.46bc	0.28 ± 0.0031bc	2.34 ± 0.38bc	86 ± 5.27	2.19 ± 0.76c
Cd0 + IONPs + SiNPs	5.89 ± 0.89a	34.98 ± 2.87a	0.56 ± 0.0039a	3.54 ± 0.39a	90 ± 5.87	3.17 ± 0.38a
Cd1 + IONPs	3.78 ± 0.37c	21.12 ± 1.78c	0.21 ± 0.0035cd	2.09 ± 0.54cd	78 ± 3.98	2.19 ± 0.57bc
Cd1 + SiNPs	2.56 ± 0.87de	18.98 ± 1.67d	0.28 ± 0.0076bc	2.56 ± 0.43c	80 ± 4.18	2.67 ± 0.18bc
Cd1 + IONPs + SiNPs	4.18 ± 0.48b	24.78 ± 1.09bc	0.39 ± 0.0072ab	2.98 ± 0.15ab	90 ± 5.28	3.08 ± 0.24ab
Cd2 + IONPs	2.76 ± 0.27d	18.23 ± 1.04de	0.17 ± 0.0043d	1.89 ± 0.76 d	72 ± 3.67	1.09 ± 0.12d
Cd2 + SiNPs	2.23 ± 0.45de	20.89 ± 1.09cd	0.26 ± 0.0021c	2.08 ± 0.45cd	80 ± 4.27	1.89 ± 0.38cd
Cd2 + IONPs + SiNPs	3.89 ± 0.48bc	23.89 ± 1.34bc	0.32 ± 0.0028b	2.65 ± 0.16c	92 ± 4.87	2.76 ± 0.17b
Cd3 + IONPs	1.89 ± 0.56e	13.76 ± 1.09f	0.17 ± 0.0021d	1.09 ± 0.78e	63 ± 3.87	0.98 ± 0.002d
Cd3 + SiNPs	2.67 ± 0.72de	15.27 ± 1.38e	0.13 ± 0.0065de	1.56 ± 0.37de	76 ± 4.76	1.67 ± 0.21cd
Cd3 + IONPs + SiNPs	3.89 ± 0.51bc	18.28 ± 1.02de	0.28 ± 0.0078bc	2.78 ± 0.28b	84 ± 5.98	2.09 ± 0.54bc

*Here IONPs, SiNPs, Cd0, Cd1, Cd2, Cd3 indicates iron oxide nanoparticles, silicon nanoparticles, 0 mM CdCl_2_, 1 mM CdCl_2_, 1.5 mM CdCl_2_, 2 mM CdCl_2_. Different letters indicate significant difference among the treatments (P ≤ 0.05).*

### Effect of Iron Oxide Nanoparticles and Silicon Nanoparticles on Leaf Relative Water Content and Net Photosynthetic Rate of *Phaseolus vulgaris*

When *P. vulgaris* seedlings were grown in non-contaminated media, combined treatment of IONPs and SiNPs enhanced LRWC content by 9 and 4% in comparison with IONPs-only and SiNPs-only treatment, respectively. A similar trend of net photosynthetic rate was observed in *P. vulgaris* seedlings exposed to different concentrations of CdCl_2_ (Table 1). IONPs and SiNPs also orchestered pigments content in *P. vulgaris* seedlings (Table 3).

**TABLE 2:** Effect of IONPs and SiNPs on root Cd content, shoot Cd content, translocation factor, and metal tolerance index of *Phaseolus vulgaris* grown in different concentrations of Cd.

Treatments	Root (μg g^–1^ DW)	Shoot (μg g^–1^ DW)
Cd0 + IONPs	ND	ND
Cd0 + SiNPs	ND	ND
Cd0 + IONPs + SiNPs	ND	ND
Cd1 + IONPs	0.013 ± 0.002	0.05 ± 0.0002
Cd1 + SiNPs	0.014 ± 0.003	0.04 ± 0.0001
Cd1 + IONPs + SiNPs	0.005 ± 0.004	0.002 ± 0.0003
Cd2 + IONPs	0.017 ± 0.001	0.009 ± 0.0001
Cd2 + SiNPs	0.015 ± 0.003	0.007 ± 0.0004
Cd2 + IONPs + SiNPs	0.019 ± 0.008	0.005 ± 0.0002
Cd3 + IONPs	0.021 ± 0.005	0.003 ± 0.0005
Cd3 + SiNPs	0.041 ± 0.0003	0.006 ± 0.0007
Cd3 + IONPs + SiNPs	0.049 ± 0.002	0.029 ± 0.0006

*Here IONPs, SiNPs, Cd0, Cd1, Cd2, Cd3 indicates iron oxide nanoparticles, silicon nanoparticles, 0 mM CdCl_2_, 1 mM CdCl_2_, 1.5 mM CdCl_2_, 2 mM CdCl_2_.*

**TABLE 3:** Effect of IONPs and SiNPs on carotenoids, total chlorophyll content, Chlb, and Chla of *Phaseolus vulgaris* grown in different concentrations of Cd.

Treatments	Carotenoids	Total Chlorophyll	Chlb	Chla
Cd0 + IONPs	4.87 ± 0.89ab	1.42 ± 0.071a	0.55 ± 0.032ab	0.87 ± 0.043
Cd0 + SiNPs	3.78 ± 0.76b	1.10 ± 0.032bc	0.34 ± 0.026c	0.76 ± 0.031
Cd0 + IONPs + SiNPs	4.93 ± 0.51a	1.19 ± 0.024bc	0.65 ± 0.016a	0.54 ± 0.017
Cd1 + IONPs	3.23 ± 0.47bc	0.81 ± 0.027c	0.35 ± 0.034bc	0.46 ± 0.026
Cd1 + SiNPs	3.02 ± 0.39c	0.66 ± 0.046	0.28 ± 0.026cd	0.38 ± 0.027
Cd1 + IONPs + SiNPs	3.16 ± 0.23bc	1.3 ± 0.037b	0.41 ± 0.017b	0.89 ± 0.031
Cd2 + IONPs	3.09 ± 0.42bc	0.78 ± 0.045c	0.31 ± 0.015cd	0.47 ± 0.025
Cd2 + SiNPs	2.89 ± 0.25cd	0.65 ± 0.067cd	0.23 ± 0.019cd	0.42 ± 0.027
Cd2 + IONPs + SiNPs	3.87 ± 0.31ab	0.96 ± 0.047bc	0.37 ± 0.023bc	0.59 ± 0.015
Cd3 + IONPs	2.78 ± 0.18cd	0.79 ± 0.067cd	0.24 ± 0.021cd	0.55 ± 0.036
Cd3 + SiNPs	2.67 ± 0.24d	0.65 ± 0.078cd	0.21 ± 0.054d	0.44 ± 0.045
Cd3 + IONPs + SiNPs	3.54 ± 0.26bc	1.13 ± 0.037ab	0.38 ± 0.018bc	0.75 ± 0.029

*Here IONPs, SiNPs, Cd0, Cd1, Cd2, Cd3 indicates iron oxide nanoparticles, silicon nanoparticles, 0 mM CdCl_2_, 1 mM CdCl_2_, 1.5 mM CdCl_2_, 2 mM CdCl_2_. Different letters indicate significant difference among the treatments (P ≤ 0.05).*

### Effect of Iron Oxide Nanoparticles and Silicon Nanoparticles on Gas Exchange Parameters of *Phaseolus vulgaris*

**FIGURE 1:**
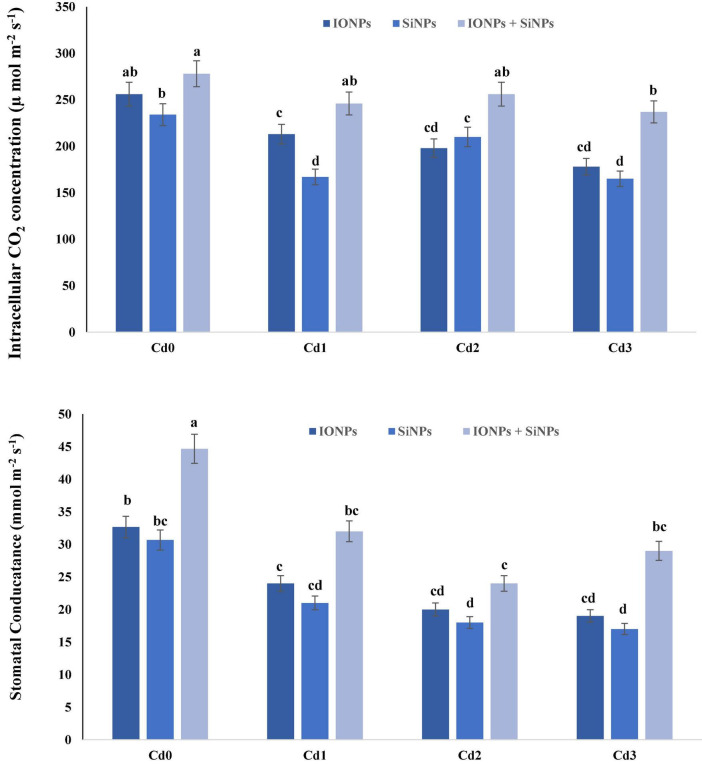
Effect of IONPs and SiNPs on intercellular CO_2_ concentration and stomatal conductance of *Phaseolus vulgaris* grown in different concentrations of Cd. Here IONPs, SiNPs, Cd0, Cd1, Cd2, and Cd3 indicate iron oxide nanoparticles, silicon nanoparticles, 0 mM CdCl_2_, 1 mM CdCl_2_, 1.5 mM CdCl_2_, and 2 mM CdCl_2_. Different letters indicate significant difference among the treatments (*P* ≤ 0.05).

In the case of *P. vulgaris* seedlings grown in non-contaminated soil, synergistic application of IONPs and SiNPs increased intercellular CO_2_ concentration and stomatal conductance by 8 and 18%, respectively, in comparison with alone application of IONP and SiNP treatment. When *P. vulgaris* seedlings were treated with 1 mM CdCl_2_, 1.5 mM CdCl_2_, and 2 mM CdCl_2_, intercellular CO_2_ concentration decreased by 20, 29, and 43%, respectively, as compared to Cd0 treatment. Synergistic application of IONP and SiNP increased intercellular CO_2_ concentration in *P. vulgaris* seedlings grown in 1.5 mM CdCl_2_ by 22 and 7%, respectively, in comparison with alone treatments of IONPs and SiNPs. Likewise, synergistic application of IONP and SiNP enhanced stomatal conductivity in *P. vulgaris* seedlings grown in Cd0-treatment and Cd-contaminated soil (Figure 1).

### Effect of Iron Oxide Nanoparticles and Silicon Nanoparticles on Malondialdehyde Content and Electrolyte Leakage in *Phaseolus vulgaris*

**FIGURE 2:**
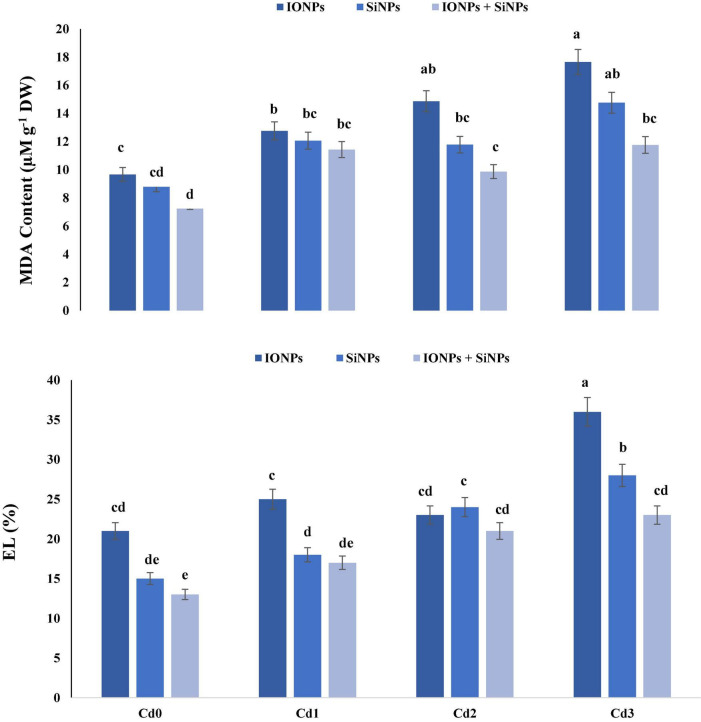
Effect of IONPs and SiNPs on MDA content and EL of *Phaseolus vulgaris* grown in different concentrations of Cd. Here IONPs, SiNPs, Cd0, Cd1, Cd2, and Cd3 indicate iron oxide nanoparticles, silicon nanoparticles, 0 mM CdCl_2_, 1 mM CdCl_2_, 1.5 mM CdCl_2_, and 2 mM CdCl_2_. Different letters indicate significant difference among the treatments (*P* ≤ 0.05).

Figure 2 shows that the application of IONPs and SiNPs reduced MDA content and EL leakage in *P. vulgaris* seedlings grown in non-contaminated and Cd-polluted soil. Synergistic application of IONPs and SiNPs reduced MDA content by 50 and 20% as compared to individual application of IONPs and SiNPs, respectively, in *P. vulgaris* seedlings grown in 1.5 mM CdCl_2_-contaminated soil.

Electrolyte leakage was also reduced in *P. vulgaris* seedlings treated with combined application of IONPs and SiNPs, in comparison with individual treatments. In the case of *P. vulgaris* seedlings grown in 2 mM CdCl_2_, combined application of IONPs and SiNPs reduced EL by 56 and 21%, respectively, in comparison with individual treatments of IONPs and SiNPs (Figure 2).

### Effect of Iron Oxide Nanoparticles and Silicon Nanoparticles on Polyamine Content in *Phaseolus vulgaris* Seedlings

**FIGURE 3:**
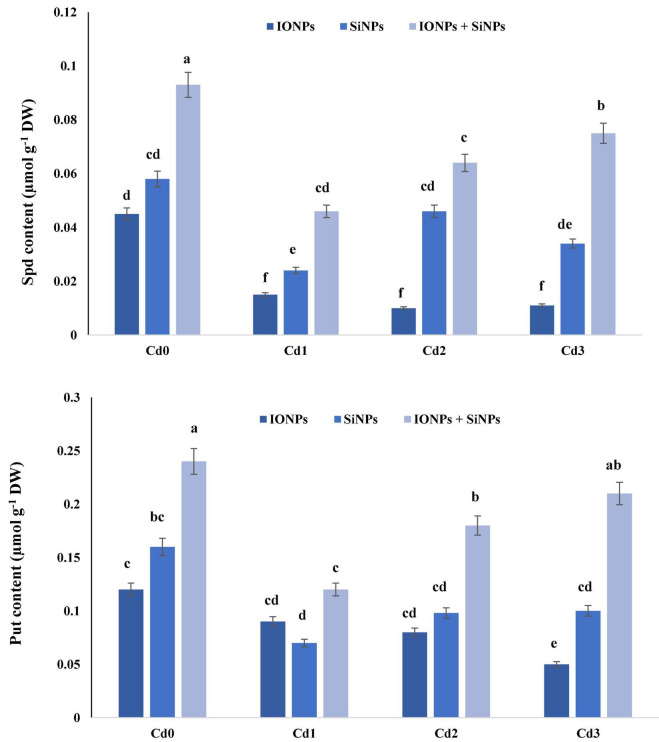
Effect of IONPs and SiNPs on PAs content of *Phaseolus vulgaris* grown in different concentrations of Cd. Here IONPs, SiNPs, Cd0, Cd1, Cd2, and Cd3 indicate iron oxide nanoparticles, silicon nanoparticles, 0 mM CdCl_2_, 1 mM CdCl_2_, 1.5 mM CdCl_2_, and 2 mM CdCl_2_. Different letters indicate significant difference among the treatments (*P* ≤ 0.05).

Figure 3 shows the effect of IONPs and SiNPs on spermidine (Spd) and putrescine (Put) content of *P. vulgaris* seedlings grown in normal and Cd-contaminated soil. Application of IONPs and SiNPs, alone or in combination, escalated Spd and Put content in *P. vulgaris.* Combined treatment of IONPs and SiNPs enhanced Spd content by 91% as compared to individual application of SiNPs in *P. vulgaris* seedlings grown in 1 mM CdCl_2_. Likewise, Put content also increased when *P. vulgaris* seedlings were treated with IONPs and SiNPs in combination. The combined application of IONPs and SiNPs enhanced Put content by more than onefold in comparison with SiNPs-only treatment in *P. vulgaris* seedlings grown in 2 mM CdCl_2_.

### Effect of Iron Oxide Nanoparticles and Silicon Nanoparticles on Nitric Oxide and Proline Content of *Phaseolus vulgaris*

**FIGURE 4:**
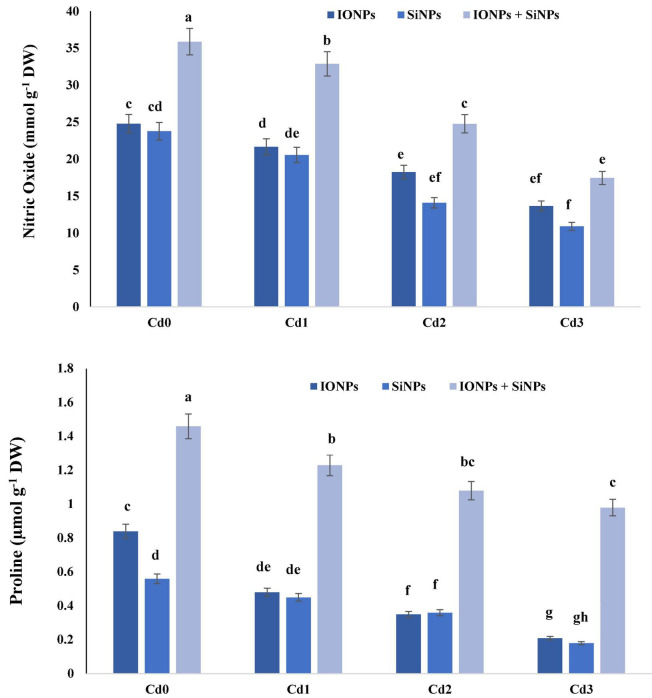
Effect of IONPs and SiNPs on nitric oxide and proline content of *Phaseolus vulgaris* grown in different concentrations of Cd. Here IONPs, SiNPs, Cd0, Cd1, Cd2, and Cd3 indicate iron oxide nanoparticles, silicon nanoparticles, 0 mM CdCl_2_, 1 mM CdCl_2_, 1.5 mM CdCl_2_, and 2 mM CdCl_2_. Different letters indicate significant difference among the treatments (*P* ≤ 0.05).

Nitric oxide and proline (Pro) content are considered as stress markers in plants. During the current study, IONPs and SiNPs enhanced nitric oxide and proline content in *P. vulgaris* seedlings grown in normal and Cd-contaminated soil. In the case of *P. vulgaris* seedlings grown in control conditions, synergistic application of IONPs and SiNPs enhanced NO content by 44 and 50%, respectively, in comparison with individual application of IONPs and SiNPs. When *P. vulgaris* seedlings were exposed to 2 mM CdCl_2_, combined application of IONPs and SiNPs enhanced NO content by more than onefold and 60% as compared to IONPs-only and SiNPs-only treatment. Similarly, IONPs and SiNPs also enhanced proline content in *P. vulgaris* seedlings grown in normal and Cd-contaminated conditions (Figure 4).

### Effect of Iron Oxide Nanoparticles and Silicon Nanoparticles on the Activity of Antioxidant Enzymes in *Phaseolus vulgaris*

**FIGURE 5:**
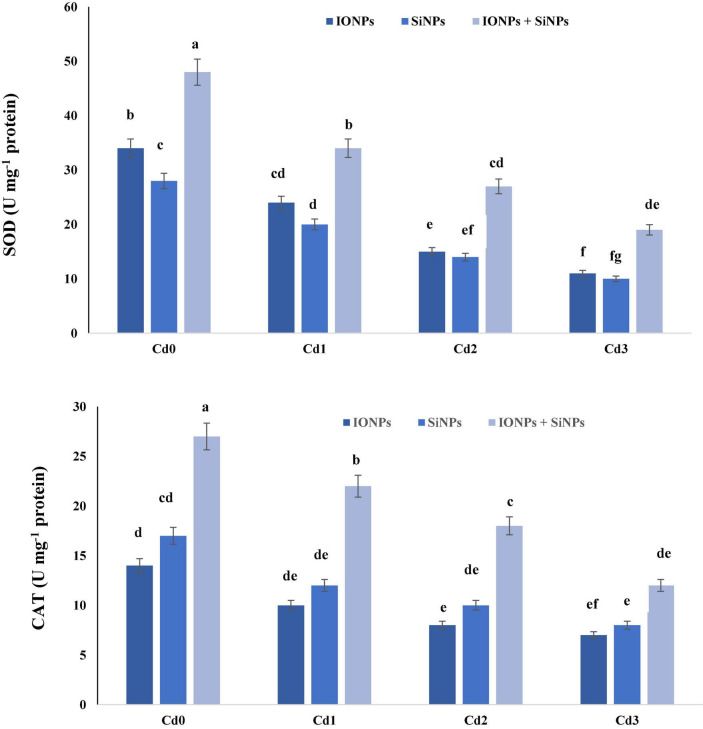
Effect of IONPs and SiNPs on SOD and CAT activity of *P. vulgaris* grown in different concentrations of Cd. Here IONPs, SiNPs, Cd0, Cd1, Cd2, and Cd3 indicate iron oxide nanoparticles, silicon nanoparticles, 0 mM CdCl_2_, 1 mM CdCl_2_, 1.5 mM CdCl_2_, and 2 mM CdCl_2_. Different letters indicate significant difference among the treatments (*P* ≤ 0.05).

Figure 5 explains the role of IONPs and SiNPs on the activity of SOD and CAT in *P. vulgaris* seedlings grown in control and Cd-contaminated conditions. In the case of *P. vulgaris* seedlings grown in Cd0 treatment, combined application of IONPs and SiNPs improved the activity of SOD by 41 and 71%, respectively, in comparison with IONPs-only and SiNPs-only treatment. Synergistic treatment of IONPs and SiNPs enhanced SOD content by 72 and 90% as compared to individual treatments of IONPs and SiNPs, respectively, in *P. vulgaris* seedlings grown in 2 mM CdCl_2_. Likewise, combined treatment of IONPs and SiNPs enhanced CAT activity in *P. vulgaris* seedlings grown in normal and CdCl_2_-contaminated conditions. The combined application of IONPs and SiNPs escalated CAT activity by 71 and 50%, as compared to IONPs-only and SiNPs-only treatment, respectively, in *P. vulgaris* seedling exposed to 2 mM CdCl_2_.

### Effect of Iron Oxide Nanoparticles and Silicon Nanoparticles on Cadmium Uptake of *Phaseolus vulgaris*

Table 2 shows that NP treatment reduced Cd uptake in *P. vulgaris* seedlings grown in CdCl_2_-contaminated soil. Synergistic application of IONPs and SiNPs reduced Cd uptake by 68% in *P. vulgaris* seedling grown in 2 mM CdCl_2._

### Effect of Iron Oxide Nanoparticles and Silicon Nanoparticles on the Nutritional Content of *Phaseolus vulgaris*

Table 4 describes the role of IONPs and SiNPs on the nutritional content of *P. vulgaris* seedlings grown in normal and different concentrations of CdCl_2_. A combined application of IONPs and SiNPs increased K^+^ content by 31 and 24%, respectively, as compared to IONPs-only and SiNPs-only treated *P. vulgaris* seedlings grown in non-contaminated potted soil.

**TABLE 4:** Effect of IONPs and SiNPs on Mo^+^, Ca^+^, K^+^, and Mn^+^ of *Phaseolus vulgaris* grown in different concentrations of Cd.

Treatments	Mo^+^ content (meq. g^–1^ DW)	Ca^+2^ content (meq. g^–1^ DW)	K^+^ content (meq. g^–1^ DW)	Mn^+2^ content (meq. g^–1^ DW)
Cd0 + IONPs	21.78 ± 1.87ab	13.56 ± 1.03b	14.87 ± 1.02bc	30.89 ± 2.56b
Cd0 + SiNPs	17.56 ± 1.08cd	15.78 ± 1.09ab	15.76 ± 1.13b	25.78 ± 1.47c
Cd0 + IONPs + SiNPs	27.98 ± 1.56a	19.87 ± 1.27a	19.56 ± 1.07a	45.98 ± 2.58a
Cd1 + IONPs	17.98 ± 1.08c	10.89 ± 1.07cd	12.56 ± 1.23cd	22.65 ± 1.45cd
Cd1 + SiNPs	15.82 ± 1.23d	11.25 ± 1.12bc	13.76 ± 1.09c	18.65 ± 1.38de
Cd1 + IONPs + SiNPs	20.56 ± 1.34b	15.89 ± 1.06ab	16.78 ± 1.56ab	24.98 ± 2.45cd
Cd2 + IONPs	13.98 ± 1.07d	10.46 ± 1.18c	09.87 ± 0.97d	14.76 ± 1.43e
Cd2 + SiNPs	14.89 ± 1.04de	11.34 ± 1.14bc	11.57 ± 1.09cd	16.34 ± 1.28de
Cd2 + IONPs + SiNPs	19.78 ± 1.37bc	12.45 ± 1.09bc	14.27 ± 1.06bc	21.59 ± 1.92d
Cd3 + IONPs	10.34 ± 1.08f	9.56 ± 1.07de	7.65 ± 0.67ef	12.98 ± 1.03ef
Cd3 + SiNPs	12.87 ± 1.17e	11.67 ± 1.56d	10.78 ± 1.08e	14. 45 ± 1.78e
Cd3 + IONPs + SiNPs	13.89 ± 1.67de	14.87 ± 1.45cd	14.87 ± 1.09de	25.89 ± 2.67c

*Here IONPs, SiNPs, Cd0, Cd1, Cd2, Cd3 indicates iron oxide nanoparticles, silicon nanoparticles, 0 mM CdCl_2_, 1 mM CdCl_2_, 1.5 mM CdCl_2_, 2 mM CdCl_2_. Different letters indicate significant difference among the treatments (P ≤ 0.05).*

## Discussion

Cadmium toxicity reduces the ability of plants to absorb key nutrients (Mg, Ca, P, K) and water content from soil (Yang et al., 2020). Potassium is considered as crucial for plant growth, development, and metabolomics (Hasanuzzaman et al., 2018) because it alleviates abiotic stresses (Raza et al., 2021) by restricting transportation and accumulation of heavy metals in plants (Kerchev et al., 2020). Additionally, potassium not only immobilizes heavy metal content (Liu et al., 2018) but also behaves antagonistically to Cd and detoxifies Cd within plants (Shamsi et al., 2008). Cadmium is a non-essential metal and is involved in the hindrance of plant growth (Shah et al. 2020a) by causing a reduction in water content, biomass, fresh and dry weight of plants, as well as disruption in redox reaction (Raza et al., 2020). Potassium application stabilizes chlorophyll architecture, which leads to the regulation of photosynthate production (Hasanuzzaman et al., 2018). It was also documented that K^+^ application enhanced growth and yield in apple dwarf rootstock seedlings (Xu et al., 2020) *via* Cd assimilation by increased accumulation of K^+^ in plants (Norvell et al., 2000). In addition, the increase in K^+^ enhanced the activity of antioxidant enzymes and reduced MDA content in plants exposed to Cd toxicity. The results of the present study also indicated that synergistic application of IONPs and SiNPs enhanced K^+^ content in *P. vulgaris* seedlings grown in normal and Cd-contaminated soil. In the present study, IONPs and SiNPs treatment increased LRWC in *P. vulgaris* seedling (Table 1). Before, it was also reported that SiNP treatment enhanced gas exchange characteristics in plants exposed to Cd-contaminated media (Ur Rahman et al., 2021). Cadmium stress destabilized photosynthetic apparatus, yet IONPs and SiNPs application reduced injury to photosynthetic machinery. Iron oxide nanoparticles increased root and shoot length in *Arachis hypogaea* plants (Rizwan et al., 2019). In the present research, it was noted also that that synergistic application of IONPs and SiNPs increased photosynthesis and gas exchange characteristics in *P. vulgaris* seedlings.

Proline is crucial for the sustenance of metabolomics in plants (Ahmad et al., 2019). It was noted in the present investigation that *P. vulgaris* seedlings treated with IONPs and SiNPs showed an increased level of proline under Cd stress. The increased level of proline in NP-treated plants might lead to activation of proline synthesizing genes involved in the regulation of water content in plants.

Membrane stability is an essential characteristic required for the effective survival of plants under abiotic stress since damage to it will lead to cellular death. MDA is an indicator of cellular damage and membrane stability (Shah et al. 2020b). Under stress conditions, the generation of MDA and peroxidation of lipids increased causing damage to plants. In this study, Cd stress amplified MDA and EL in *P. vulgaris* plants grown in Cd-contaminated soil. Cadmium decreases the stability of membranous molecules due to the enhanced accumulation of ROS. Previous researchers revealed the role of IONPs and SiNPs in alleviating abiotic stresses in plants.

The total antioxidant activity of a plant is a good indicator of how well the plant’s antioxidant system is functioning in the presence of oxidative stress. Plants have a variety of antioxidative defense systems to deal with oxidative stress, particularly those produced by the generation of excess ROS as a result of abiotic stress. Superoxide peroxidase (SOD) is the first line of defense that is involved in the conversion of superoxide anion to peroxide. Catalase (CAT) is also a crucial antioxidant that plays an important role in plants’ defensive approach to abiotic stresses. This enzyme is involved in the conversion of H_2_O_2_ to O_2_ and H_2_O. Current research reported that IONPs and SiNPs enhanced the activity of CAT in *P. vulgaris* seedlings exposed to Cd stress.

Nanoparticles reduced MDA levels, while they increased peroxidase (POD) and SOD activity in metal-stressed wheat plants (Sardar et al., 2022). Additionally, osmoregulators control the structure of organelles and macromolecules in plants by adjusting osmosis and aid in the reduction of Cd-induced toxicity in plants (Thind et al., 2021). In the present study, we also noticed that Cd inhibited the production of total soluble proteins and proline concentration in *P. vulgaris* seedlings. The findings of the present study were in line with the findings of Azizi et al. (2021). Application of NPs in the present study enhanced proline accumulation in Cd treated *P. vulgaris* seedlings. Enhanced accumulation of proline and soluble sugar may be due to augmented proline playing some vital roles in scavenging ROS, adjusting osmotic equilibrium and determining the membrane attributes in plants exposed to stress (Aswani et al., 2019). Many authors investigated the positive effect of NPs on the enhancement of proline accumulation under stress conditions in broad bean plants in addition to adjusted antioxidant enzyme activities, soluble sugars, and amino acids (Sadak and Bakry, 2020). Furthermore, Azimi et al. (2021) claimed that NP supply can decline salt stress in tomatoes by improving photosynthetic machinery, phenolics, and antioxidant enzyme activities and yield as well.

Cadmium phytotoxicity increased the formation of ROS and produced oxidative burst by interfering with the antioxidant defense system (Kamran et al., 2021), which promotes the MDA content due to the high accumulation of lipid peroxidation of the membrane. During the normal metabolism, the plants have established well-organized antioxidant enzyme defense mechanisms to eliminate the ROS (Fan et al., 2020). This is because SOD acts as the first line of defense in plants against ROS. Superoxide dismutase converts the O_2_^–^ to less toxic H_2_O_2_, and it forms the first line of defense in the antioxidant system of plants; to this end, CAT scavenges the H_2_O_2_ to water and oxygen molecules (Orabi and Abou-Hussein, 2019). The ameliorative influence of the NPs on Cd stress was also assessed by Hussain et al. (2018) in the wheat plant. This apparently will promote antioxidant impairments by decreasing H_2_O_2_ and MDA content and enhancing the activities of SOD, CAT, and increased tolerance against abiotic toxicity (Sharma et al., 2019).

## Conclusion

Cadmium toxicity enhanced oxidative stress in *P. vulgaris* seedlings. Application of IONPs and SiNPs escalated the activity of antioxidant enzymes besides incrementation in K^+^ content. Additionally, synergistic application of IONPs and SiNPs increase NO content and Pro content in *P. vulgaris* seedlings grown in normal and CdCl_2_-polluted soil. Cd-stress alleviation is credited to increased activity of antioxidant enzymes and maintenance of PAs and K^+^ metabolism. It is further added that mechanisms and molecules involved in the interaction of different NPs can be exploited for the mitigation of numerous stresses in plants.

## Data Availability Statement

The original contributions presented in the study are included in the article/supplementary material, further inquiries can be directed to the corresponding author/s.

## Author Contributions

LK contributed toward designing the research and conceptualization. AU and AAS performed the experiments. AAS performed the statistical analysis. NAY and ZS contributed to the review and drafting. LR contributed to writing the manuscript. AR and TJ drafted the manuscript. MHS and SA were involved in funding acquisition and writing. All authors contributed to the article and approved the submitted version.

## Conflict of Interest

The authors declare that the research was conducted in the absence of any commercial or financial relationships that could be construed as a potential conflict of interest.

## Publisher’s Note

All claims expressed in this article are solely those of the authors and do not necessarily represent those of their affiliated organizations, or those of the publisher, the editors and the reviewers. Any product that may be evaluated in this article, or claim that may be made by its manufacturer, is not guaranteed or endorsed by the publisher.
